# Preoperative Predictors of Biochemical Recurrence-Free Survival in High-Risk Prostate Cancer Following Radical Prostatectomy

**DOI:** 10.3389/fonc.2020.01761

**Published:** 2020-09-10

**Authors:** Gerard Nkengurutse, Feng Tian, Sixiong Jiang, Qi Wang, Ying Wang, Weibing Sun

**Affiliations:** Department of Urology, The Second Affiliated Hospital of Dalian Medical University, Dalian, China

**Keywords:** prostate cancer, high risk, radical prostatectomy, biochemical recurrence, BCR-free survival

## Abstract

**Background:** D'Amico high-risk prostate cancer (Pca) patients experience poor and heterogeneous oncological outcomes. This heterogeneity highlights a need to extensively explore factors associated with poor outcomes to guide decision-making.

**Objective:** To assess predictors of biochemical recurrence (BCR)-free survival in high-risk patients following radical prostatectomy (RP), and subsequently establish a model predicting outcomes.

**Methods:** We retrospectively identified D'Amico high-risk non-metastatic Pca patients who underwent RP between 2013 and 2019 in our hospital. We collected data including PSA level, clinical stage, biopsy Gleason score (GS), number of D'Amico high-risk factors (RF), the inflammatory status (Neutrophil-to-lymphocyte ratio [NLR], derived NLR [dNLR], platelet-to-lymphocyte ratio [PLR] and LDH). Kaplan–Meier methods were used to analyze BCR-free survival. Univariate and multivariate analyses were performed using Cox proportional hazards model to evaluate the association between clinicopathological parameters and BCR-free survival.

**Results:** The median follow-up time for the 101 patients' cohort was 26 months (range: 3–81 months). The number of RF (1RF vs. ≥2RF), biopsy GS (<8 vs. ≥8), clinical stage (≤cT2c vs. >cT2c), pathological stage, and the presence of adverse pathological features were significant predictors of BCR (*P* < 0.05). Other parameters including inflammatory status (dNLR, NLR, PLR, and LDH) were not of predictive value. On multivariable analysis, biopsy GS (<8 vs. ≥8; HR 2.439) and clinical stage (≤cT2c vs. >cT2c; HR 3.271) were the independent predictors of BCR. Based on these two independent predictors, patients were stratified into three risk subgroups: favorable (0 risk factor; 47% of patients), intermediate (1 risk factor; 42 %), unfavorable (2 risk factors; 11%). The intermediate and unfavorable subgroups have a significantly shorter median BCR-free survival compared to the favorable subgroup (*P* < 0.001).

**Conclusion:** Several factors are associated with BCR. Clinical stage (≤cT2c vs. >cT2c) and biopsy GS (<8 vs. ≥8) are the independent predictors of BCR. The stratification of high-risk patients into risk subgroups based on these two predictors shows that the intermediate and unfavorable subgroups have a significantly shorter median BCR-free survival compared to the favorable subgroup. The preoperative stratification model may help urologists and patients during decision-making. In non-metastatic high-risk patients, preoperative inflammatory markers (NLR, dNLR, PLR, and LDH) are not of prognostic value.

## Introduction

Prostate cancer (Pca) is the second most commonly diagnosed cancer in men, with an estimated 1.3 million diagnoses worldwide in 2018, accounting for 14% of all cancers (1). It is the most frequently diagnosed cancer among men in more developed countries. The incidence of Pca varies widely (~25-fold) worldwide, the highest being in Australia and New Zealand and the lowest in South-Central Asia (2, 3).

It is the fifth leading cause of cancer death in men and the worldwide Pca burden is expected to grow to 1.7 million new cases and 499,000 new deaths by 2030 due to in part to the growth and aging of the population in addition to environmental factors. The highest mortality rates are found in the Caribbean and Southern and Middle Africa (2). In 2008, the estimated number of prostate cancer deaths in sub-Saharan Africa (SSA) was more than five times that of African Americans and is expected to double in Africa by 2030. Interestingly, while the mortality rate is decreasing in Northern America, Oceania, and Northern and western Europe, it is increasing in Asian, and central and eastern European countries such as China, Korea, and Russia (4). Risk factors associated with economic development— increased consumption of animal fat, obesity, and physical inactivity—are thought to be the reason for this increase (4).

D'Amico et al. (5) classified Pca patients into risk groups based on clinical and pathologic parameters, allowing predicting relapse risk before treatment. Patients with PSA ≥ 20 ng/ml, clinical stage of ≥T2c or biopsy Gleason score (GS) ≥8 are considered to be at high risk. Since then, many definitions of high-risk patients have been proposed and are being used in clinical practice (6).

Different guidelines recommend radical prostatectomy (RP) either open (ORP), laparoscopic (LRP), or robot-assisted (RARP) as the treatment modality for low-risk and intermediate-risk young patients with acceptable comorbidities. However, there is no consensus regarding the optimal treatment of men with high-risk Pca. RP can be performed with curative intent. Radiation therapy plus androgen deprivation therapy (ADT) has also been shown to provide similar long-term cancer control for patients with high-risk disease compared to RP (7).

Therefore, high-risk prostate cancer treatment remains controversial. Despite the feasibility and safety of RP in high-risk prostate cancer patients, some patients still experience biochemical recurrence (BCR), progression and metastasis after the operation. For example, depending on the definition used, the 5-year BCR-free survival can range from 49 to 80% (8). Moreover, it has been shown that not all high-risk Pca patients have a uniformly poor prognosis after RP (9).

This heterogeneity in outcomes in this patient group shows that there is a need to explore extensively the factors associated with poor outcomes to guide decision making during the choice of the optimal treatment.

A number of researchers have attempted to substratify this patient group in order to guide optimal treatment decision-making (8–14). Some report favorable outcomes in patients with one D'Amico risk factor compared to those with two or more risk factors (8–10, 14) while others add new parameters like percentage of positive cores (13), thus leading to different scoring systems.

Inflammation is now widely acknowledged to be involved in cancer development, progression and metastasis. Rudolf Virchow—by observing the presence of leukocytes within tumors in 1863—provided insights of a possible link between inflammation and cancer (15). The host response in the form of systemic inflammation has been shown as an independent predictor of oncological outcomes (16). Moreover, the combination of hematological components of the systemic inflammatory response, such as the neutrophil–lymphocyte ratio (NLR), derived neutrophil-to-lymphocyte ratio (dNLR), platelet-to-lymphocyte ratio (PLR), has been reported to be of prognostic value in several types of cancer (16, 17). However, the findings are inconclusive, contradictory or of weak evidence, particularly in early stage and less aggressive disease. High-risk Pca can be sometimes of both early stage and more aggressiveness. In prostate cancer, most studies showed a prognostic value of these markers in a metastatic disease (especially castration-resistant prostate cancer: CRPC) (18), with only a few studies on a localized disease and showing contradictory results (19, 20). Therefore, preoperative peripheral inflammation-based markers—if of prognostic value—can be incorporated in the risk stratification of high-risk Pca patients, helping in the decision-making about the optimal treatment.

Our main objective was to assess prognostic factors of biochemical recurrence (BCR)-free survival in high-risk non-metastatic Pca patients following radical prostatectomy (RP), and subsequently establish a model predicting outcomes. Such a model can help identify patients who benefit the most from RP alone, thus aiding urologists and patients to reach a shared clinically important decision-making regarding the choice of the optimal treatment.

## Patients and Methods

### Patient Population

This is a retrospective, unicenter study in which data on prostate cancer patients who underwent ORP or LRP or RARP between January 2013 and September 2019 were collected from medical records at the 2nd Hospital of Dalian Medical University. Informed consent from patients was waived, given the retrospective nature of the study.

We reviewed the medical records of the 872 patients who were diagnosed with prostate cancer to detect high-risk ones who underwent radical prostatectomy. Staging was in accordance with the 2002 TNM classification for adenocarcinoma of the prostate. Among 293 radical prostatectomies that were performed, we proceeded to identify high-risk patients according to D'Amico risk classification system. One hundred and one patients met our inclusion criteria. All these patients underwent preoperative cross-sectional abdominopelvic imaging (abdominal CT and pelvic MRI) and a bone scan to rule out visceral or bone metastasis. Only one patient underwent PSMA PET/CT after abdominopelvic CT and a bone scan because he was relatively young (54 years of age) with a high Gleason grade (5+4) and PET/CT was expected to change the treatment regimen if a systemic disease is discovered.

One hundred patients underwent transrectal ultrasound (TRUS)-guided prostate biopsy and one underwent MRI-TRUS fusion target biopsy. Seventy-nine patients underwent systematic TRUS-guided biopsy; 16 patients with MRI-suspected prostate lesions underwent both cognitive registration TRUS targeted biopsy and systematic biopsy. The median and mean of the number of cores were 12 and 11.51, respectively. Data on cores number of five patients biopsied in another hospital were missing.

### Inclusion and Exclusion Criteria

Our inclusion criteria were predefined as follows:

1. High-risk Pca based on D'Amico risk classification:
  - PSA ≥ 20 ng/ml, AND/OR
  - Clinical stage of ≥T2c, AND/OR
  - Biopsy Gleason score (GS) ≥8
2. Having undergone Radical prostatectomy as treatment modality;
3. Having undergone bone scintigraphy (scan) and other imaging tests to rule out a metastatic disease.

We excluded the patients in the following scenarios:

- Patients who underwent RP despite a proof of a metastatic disease;
- Patients who received neoadjuvant hormonal therapy;
- Patients without complete clinical and pathological data.

### Data Collection

For the included patients, de-identified preoperative and postoperative data were collected. Preoperative data included patient age, preoperative serum PSA level, clinical stage, biopsy GS, number of high risk factors (RF) based on D'Amico risk classification system, number of cancer-positive cores, percentage of positive biopsy cores and the inflammatory status (White blood cell count [WBC], absolute neutrophil count [ANC], absolute lymphocyte count [ALC], absolute platelet count [APC] and lactate dehydrogenase level [LDH]). Neutrophil-to-lymphocyte ratio (NLR), derived Neutrophil-to-lymphocyte ratio (dNLR), platelet-to-lymphocyte ratio (PLR) were subsequently calculated: dNLR = ANC/(WBC-ANC); NLR = ANC/ALC; PLR = APC/ALC. Postoperative information included the GS of the specimen, the pathological stage, the presence of extraprostatic extension (EPE) or seminal vesicle invasion (SVI), lymph node invasion (LNI), and the surgical margins status (R0 vs. R1). NLR, dNLR, and PLR were defined as high if they were superior to their respective medians (2.023474; 1.486928; 120.4188, respectively). Lactate dehydrogenase (LDH) levels were considered high if they were greater than the upper limit of normal (ULN).

### Outcomes Assessment

The primary endpoint was the biochemical recurrence to allow calculation of biochemical recurrence-free survival (BCR-free survival) after RP. Preoperative serum PSA levels were measured within 1 month of RP, and all patients underwent follow-up assessment of PSA, testosterone level, bone scintigraphy, and other imaging tests (abdominopelvic CT or pelvic MRI and chest CT). Most patients' PSA and testosterone levels were taken 1 month postoperatively, then every 3 months for the first 2 years post-treatment, and every 6 months thereafter. BCR was defined as PSA level > 0.2 ng/ml with two consecutive increases. If PSA levels did not decrease to <0.2 ng/mL after surgery, then the date of RP was defined as the date of disease recurrence.

### Statistical Analysis

The Kaplan–Meier method and the log-rank test were used to estimate the statistical differences in BCR-free survival. Univariate analysis was performed using the Cox proportional hazards regression model for evaluation of the association between clinicopathological parameters and BCR-free survival. Taking clinical background into account and *P*-value of 0.1 or less on univariate analysis, multivariate analysis was performed using the Cox proportional hazards model, for the estimation of the factors predicting BCR-free survival. *P* < 0.05 was considered to be of statistical significance. Data were first collected in Microsoft Excel. Then, the Statistical Package for the Social Sciences Version 25.0 (IBM SPSS Statistics for Windows, Version 25.0. Armonk, NY: IBM) was used for all statistical analyses.

## Results

### Patient Demographics

The median follow-up time was 26 months (range: 3–81 months) and the median age at surgery was 71 years (range: 51–85 years). Of 101 patients that met the inclusion criteria, 49 patients had only one high-risk factor (RF) while 52 had two or more RFs according to D'Amico risk classification. Seventy-nine patients had a localized disease (≤cT2c) and 22 patients have >cT2c. Fourty-two patients had biopsy GS ≥ 8 and 72 patients had a PSA ≥ 20 ng/ml. BCR occurred in 45% of patients. Most of patients received adjuvant ADT. Clinicopathological characteristics of the study patients are described in Table 1.

**Table 1:** Clinicopathological characteristics of the study patients.

	***n* (%)**
Mean (range) age (in years)	71 (51–85)
Median (range) follow-up time (in months)	26 (3-81)
**Clinical T Stage**	
<cT2c	40 (39.6)
≥cT2c	61 (60.4)
≤cT2c	79 (78.2)
>cT2c	22 (21.8)
**Preoperative PSA**	
<20 ng/ml	29 (28.7)
≥20 ng/ml	72 (71.3)
**GS at biopsy**	
<8	59 (58.4)
≥8	42 (41.6)
<9	81 (80.2)
≥9	20 (19.8)
**Gleason pattern 5 at biopsy**	
No Gleason pattern 5	78 (77.2)
Presence of Gleason pattern 5	23 (22.8)
**Number of risk factors (RF) based**	
**on D'Amico classification**	
1RF	49 (48.5)
≥2RF	52 (51.5)
**Number of positive cores**	
≤3	18 (17.8)
>3	78 (77.2)
Missing	5 (4.95)
**% of positive cores**	
≤33%	27 (26.7)
>33%	57 (56.4)
Missing	17 (16.8)
**Inflammatory status**	
dNLR (classified as High or Low)	
High	51 (50.5)
Low	50 (49.5)
NLR (classified as High or Low)	
High	50 (49.5)
Low	51 (50.5)
PLR (classified as High or Low)	
High	51 (50.5)
Low	50 (49.5)
LDH (classified as High or Low)	
High	18 (17.8)
Low	82 (81.2)
Missing	1 (≈1)
**Pathological features**	
GS of the specimen	
<8	47 (46.5)
≥8	54 (53.5)
≥9	40 (39.6)
<9	61 (60.4)
No Gleason pattern 5	57 (56.4)
Presence of Gleason pattern 5	44 (43.6)
Pathological stage	
Organ-confined	43 (42.6)
Non-organ confined	58 (57.4)
EPE or SVI	
0	49 (48.5)
1	52 (51.5)
Lymph node invasion	
0	60 (59.4)
1	19 (18.8)
Missing	21 (20.8)
Surgical margin status	
R0	57 (56.4)
R1	43 (42.6)
Missing	1 (≈1)

### Analysis of BCR-Free Survival

**Figure 1:**
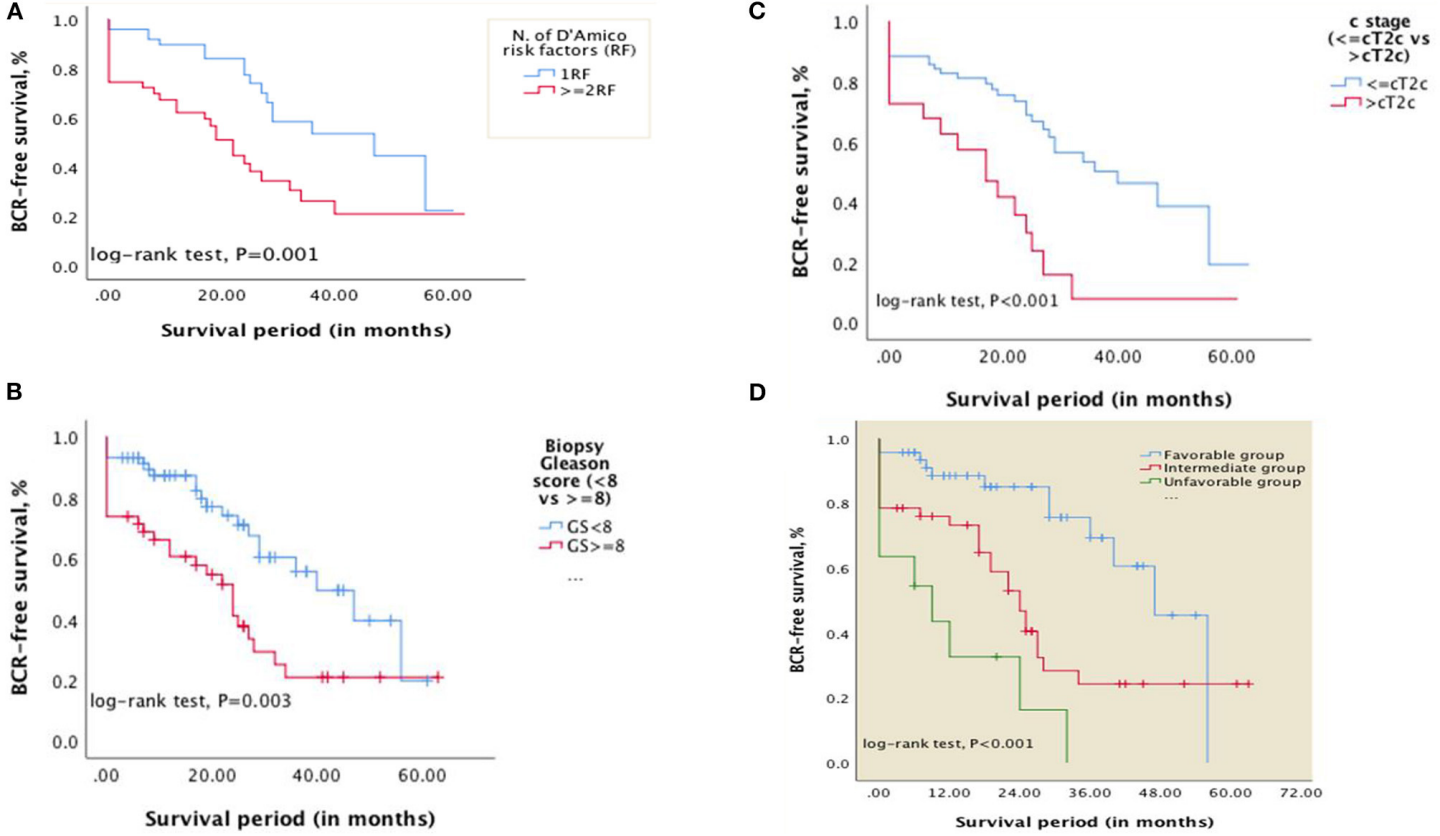
Kaplan–Meier curves showing differences in BCR-free survival in patients with: **(A)** D'Amico RF (1RF vs. ≥2RF); **(B)** biopsy Gleason score <8 vs. ≥8; **(C)** clinical stage ≤cT2c vs. >cT2c. **(D)** Kaplan–Meier curves showing differences in BCR-free survival in patients categorized into risk subgroups (Favorable, intermediate, and unfavorable) based on the number of risk factors—Gleason score at biopsy (<8 vs. ≥8) and clinical stage (≤cT2c vs. >cT2c) (*P* < 0.001).

The overall median BCR-free survival was 29 months [standard error (SE): 3.643; 95% CI, 21.859–36.141]. The 2, 3, and 5-year BCR-free survival were 64.4, 39.6, and 21%, respectively. For patients with one D'Amico RF, the median BCR-free survival was 47 months [SE: 14.830; 95% CI: 17.933–76.067] while it was 22 months (SE: 2.653; 95% CI: 16.800–27.200) for those with ≥2 D'Amico RF (Figure 1A).

On univariate analysis, the number of D'Amico high-risk factors (1RF vs. ≥2RF), Gleason score (GS) at biopsy (<8 vs. ≥8; <9 vs. ≥9; or presence of Gleason component 5), clinical T stage (≤cT2c vs. >cT2c), GS of the specimen (GS <8 vs. ≥8), pathological T stage (organ-confined vs. non organ-confined), surgical margin status (R0 vs. R1) and the presence of EPE or SVI were predictors of BCR (*P* < 0.05) (see Table 2). The age, preoperative PSA level (<20 vs. ≥20 ng/ml), clinical stage (<cT2c vs. ≥cT2c), LNI, inflammatory status (dNLR, NLR, PLR, and LDH); number of positive cores, percentage of positive cores were not of predictive value (*P* > 0.05). On multivariate Cox regression analysis, organ-confined vs. non organ-confined disease (i.e., pT stage) was excluded from explanatory variables for correlation because SVI and EPE strongly correlate with pT stage; the number of D'Amico risk factors was also excluded since it correlates with biopsy GS and clinical stage. Biopsy Gleason pattern 5 and GS ≥ 9 were excluded as well since they strongly correlate with GS ≥ 8. GS at biopsy [<8 vs. ≥8, HR 2.439; 95% CI, 1.110–5.361; *P* = 0.026] and clinical stage [≤cT2c vs. >cT2c; HR 3.271; 95% CI, 1.425–7.510; *P* = 0.005] were the independent predictors of BCR (see Table 2).

**Table 2:** Univariate and multivariate analysis of predictors of BCR.

**Factor**	**Univariate**			**Multivariate**		
	**HR**	**95% CI**	***P*-value**	**HR**	**95% CI**	***P*-value**
Age (≤65 vs. >65)	0.769	0.402–1.469	0.426			
PSA in ng/ml (<20 vs. ≥20)	1.311	0.690–2.489	0.408	0.984	0.426–2.273	0.970
Clinical stage (<cT2c vs. ≥cT2c)	1.493	0.821–2.716	0.189			
(≤cT2c vs. >cT2c)	2.796	1.523–5.132	**0.001**	3.271	1.425–7.510	**0.005**
**GS at biopsy**						
(<8 vs. ≥8)	2.296	1.284–4.105	**0.005**	2.439	1.110–5.361	**0.026**
Gleason pattern 5 (1 vs. 0)	2.611	1.445–4.720	**0.001**			
(<9 vs. ≥9)	2.437	1.329–4.468	**0.004**			
Number of D'Amico RF (<2RF vs. ≥2RF)	2.528	1.383–4.621	**0.003**			
Number of positive cores (≤3 vs. >3)	2.581	0.923–7.221	0.071	1.718	0.363–8.132	0.495
% of positive cores (≤33 vs. >33)	2.010	0.923–4.378	0.079	0.976	0.403–2.359	0.956
**Inflammatory status**						
dNLR (Low vs. High)	0.751	0.420–1.345	0.336			
NLR (Low vs. High)	1.131	0.633–2.021	0.678			
PLR (Low vs. High)	1.020	0.575–1.811	0.946			
LDH (Low vs. High)	0.449	0.188–1.076	0.072	0.375	0.128–1.101	0.074
GS of the specimen						
<8 vs. ≥8	3.052	1.618–5.759	**0.001**			
**Pathological stage**						
Organ-confined vs. Non Organ-confined	2.575	1.353–4.902	**0.004**			
EPE or SVI (0 vs. 1)	2.329	1.267–4.284	**0.007**	1.458	0.570–3.732	0.431
LNI (0 vs. 1)	1.936	0.957–3.914	0.066	0.890	0.341–2.319	0.811
Surgical margins (R0 vs. R1)	1.866	1.043–3.336	**0.035**	1.091	0.487–2.444	0.832

*Bold values are statistically significant P < 0.05*.

Using Kaplan-Meier curves and log-rank tests, differences in BCR-free survival were calculated (Figures 1B,C). The median BCR-free survival for patients with biopsy GS <8 vs. GS ≥8 was 40 months [SE 6.687; 95% CI, 26.894–53.106] and 24 months [SE:3.565; 95% CI, 17.013–30.987], respectively (*P* = 0.003). For patients with clinical stage ≤cT2c vs. >cT2c, the BCR-free survival was 40 months [SE: 7.960; 95% CI, 24.398–55.602] and 17 months [SE:4.969;95% CI, 7.260–26.740], respectively (*P* < 0.001).

### Preoperative Risk Stratification

Based on the two independent predictors on multivariate analysis, patients were categorized into three risk subgroups as follows: favorable (0RF, i.e., biopsy Gleason score <8 and clinical stage ≤cT2c and any PSA level; 47%), intermediate (1RF, i.e., biopsy Gleason score ≥8 or clinical stage >cT2c and any PSA level; 42 %,), unfavorable (2RF, biopsy Gleason score ≥8 and clinical stage >cT2c and any PSA level; 11%). The 1 and 2-year BCR-free survival were 88.6 and 85.1% for the favorable group, 73.3 and 53.1% for the intermediate group, and 32.7 and 16.4% for the unfavorable group, respectively. The median BCR-free survival for favorable, intermediate and unfavorable subgroups was 47 months [SE: 4.161; 95% CI, 38.845–55.155], 24 months [SE: 2.728; 95% CI, 18.654–29.346], and 9 months [SE: 3.942; 95% CI, 0.000–22.648], respectively (*P* < 0.001) (See Figure 1D).

## Discussion

High-risk Pca is known to be associated with poor outcomes and no consensus about the optimal treatment modality is available. However, it has been shown that not all high-risk Pca patients have a uniformly poor prognosis after RP (9). This heterogeneity in outcomes in this patient group shows that there is a need to explore extensively the factors associated with poor outcomes to guide decision-making during the choice of the optimal treatment.

In the present study, we showed that the Gleason score (GS) at biopsy (<8 vs. ≥8) and clinical stage (≤cT2c vs. >cT2c) are independent predictors of BCR in high-risk Pca patients. We subsequently subcategorized these patients into favorable (0 RF), intermediate (1 RF), and unfavorable subgroups (2 RF) based on these two prognosticators. The 1 and 2-year BCR-free survival were 88.6 and 85.1% for the favorable group, 73.3 and 53.1% for the intermediate group, and 32.7 and 16.4% for the unfavorable group, respectively. From a clinical standpoint, this information may help urologists and patients during a shared decision making process. High-risk Pca patients experience heterogeneous outcomes after RP and efforts to stratify these patients into risk subgroups have been made (8–14), mostly reporting that high-risk Pca patients with one D'Amico high risk factor have better outcomes compared to those with ≥2 risk factors. However, such useful classification can also miss important information since it will seem that, for instance, a patient with PSA ≥ 20 ng/ml (i.e., one D'Amico RF) has the same outcomes as one who has GS ≥ 8 (i.e., one D'Amico RF too).

In our study, dichotomization of PSA (<20 vs. ≥20 ng/ml) didn't provide significant information for the prediction of BCR-free survival. Many explanations are possible; PSA is not a dichotomous marker but one whose values reflect a continuum of risk for prostate cancer (21). In addition, PSA is not specific for malignancy and can be elevated due to other conditions such as benign prostatic hyperplasia (BPH), prostatitis, urinary retention or physical manipulation. Indeed, it has been reported that PSA is the least valuable predictor (6, 8, 11, 22), and 20 ng/ml as the optimal threshold to define a high risk for BCR-free survival or long-term oncological outcomes is a matter of debate. Our study corroborates these results. The substratification model proposed by Joniau et al. (8) was not significant for BCR-free survival; and Beauval et al. (10) also didn't find PSA as an independent predictor of BCR-free survival although they conclude that RP provides both effective cancer control and satisfactory survival rates in patients with only one D'Amico risk factor.

D'Amico high-risk group is widely acknowledged to be heterogeneous. The European Association of Urology (EAU) risk group classification—which is essentially based on D'Amico's classification system— acknowledges this heterogeneity by further dividing this group into a localized disease (cT2c) and locally advanced disease (cT3-4 or cN+) (23). The National Comprehensive Cancer Network (NCCN) risk stratification for Pca incorporates clinical stage cT2c into the intermediate-risk group, even recognizing that men assigned to the intermediate-risk group by clinical stage (T2b–T2c) have a lower risk of recurrence than men categorized according to GS of 7 (24). Our findings are in line with these guidelines. In fact, dichotomization of our patients based on <cT2c vs. ≥cT2c for the BCR-free survival didn't show a significant prognostic value (*P* > 0.05) while ≤cT2c vs. >cT2c provided a significant predictive value for BCR-free survival on both univariate and multivariate analysis (Table 2).

Our results not only support a risk stratification based on number of high-risk features but also specifies these. The favorable subgroup (i.e., 0 RF) in our model includes these three types of patients: (a) PSA ≥ 20 ng/ml and clinical stage <cT2c and biopsy GS <8; (b) PSA <20 ng/ml and clinical stage =cT2c and biopsy GS <8; and (c) PSA ≥ 20 ng/ml and clinical stage =cT2c and biopsy GS <8. Our substratification supports previous studies reporting that patients with one D'Amico high-risk factor experience better outcomes. The median BCR-free survival for our favorable subgroup (Figure 1D) and the median BCR-free survival in patients with only one D'Amico RF (Figure 1A) were similar: 47 months [SE: 4.161; 95% CI, 38.845–55.155] and 47 months [SE: 14.830; 95% CI: 17.933–76.067], respectively.

More importantly, in contrary to many prior reports that high-risk Pca patients with one D'Amico high-risk factor experience better outcomes compared to those with ≥2 risk factors, our model suggests that patients with two D'Amico high-risk factors can also have good outcomes in this scenario: cT2c and PSA ≥ 20 ng/ml. However, these patients actually have only one high-risk factor according to NCCN risk stratification.

The present categorization into risk subgroups is different from models in prior studies in the fact that patients with biopsy GS ≥ 8 (i.e., just one D'Amico high-risk factor) are not part of the favorable subgroup. In fact, the risk of recurrence appears to be very high in patients with high-grade disease (10). While one may consider that biopsy GS = 8 (i.e., 4 + 4 = 8, 3 + 5 = 8, 5 + 3 = 8) as a single D'Amico high-risk factor is a favorable feature, heterogeneity in cancer control has been reported amongst men with GS 3 + 5/5 + 3 vs. GS 4 + 4 (25, 26). Taking into account the paucity of evidence of this heterogeneity in terms of BCR-free survival and the fact that Gleason grade is the feature that more accurately and more clearly reflects tumor aggressiveness (27), patients with GS ≥ 8 (even as a single D'Amico high-risk factor) should not be considered as a favorable subgroup, especially when the Gleason pattern 5 is present.

Inflammation is known to be associated with the development and progression of cancer. The prognostic value of hematological markers of the systemic inflammatory response in cancer patients has garnered a lot of interest in the last decade, with contradictory or of weak evidence findings, particularly in early stage and less aggressive disease (16). Taking into account that high-risk Pca can be of both early stage and more aggressiveness, we investigated the prognostic value of dNLR, NLR, PRL, and LDH on BCR-free survival. None of these were found to be a significant prognosticator on univariate analysis (*P* > 0.05). Our results are in line with the widely acknowledged hypothesis that peripheral inflammation-based markers are strong prognostic factors of oncological outcomes in advanced disease (16). Moreover, our findings are very similar to those of Zanaty et al. (28) and Bahig et al. (29) who reported in their recent studies that NLR and PLR were not significant predictors of BCR even on univariate analysis. Another challenge in implementing these factors in clinical practice is that there is no consensus on a validated standard cut-off value for these scores. For example, in the systematic review on the prognostic value of NLR in Pca by Cao et al. (30), an elevated NLR in many studies was defined as NLR ≥ 5 or NLR ≥ 3 while some defined NLR ≥ 2 as an elevated one, thus varying from 2 to 5. To our knowledge, no study has been conducted to specifically investigate the prognostic value of inflammation-based scores in high-risk Pca patients. We hypothesized that high-risk Pca constitutes a very aggressive disease and that, therefore, peripheral inflammation-based markers may be of strong prognostic value in this patient group. Although two meta-analyses reported NLR to be of prognostic value in localized Pca (18, 30), recent studies continue to find contradictory results (28, 29, 31).

This study has several limitations. First, the retrospective nature of the study has its inherent limitations. The sample size is relatively small to allow powerful conclusions. Further and more powered studies are needed to validate the findings. Second, in some patients, the performed pelvic lymph node dissection was not the extended one (ePLND) that is now recommended in high-risk Pca patients. This may constitute a major confounder for measured outcomes. Moreover, although most patients were treated with laparoscopic radical prostatectomy plus ePLND (LRP + ePLND), some patients were treated with open RP and three patients were treated with robot-assisted radical prostatectomy (RARP). This difference may bring to different oncological outcomes. That said, according to a Cochrane systematic review in 2017, there is no high-quality evidence to inform the comparative effectiveness of LRP or RARP compared to ORP for oncological outcomes (32).

## Conclusion

A number of preoperative and postoperative factors are associated with BCR-free survival. Our results identified clinical T stage (≤cT2c vs. >cT2c) and Gleason score of the biopsy (<8 vs. ≥8) to be independent predictors of BCR. The new categorization of high-risk Pca patients into risk subgroups (favorable, intermediate and unfavorable) based on these predictors show that patients with 1 RF and 2 RF have a much shorter median biochemical recurrence-free survival compared with those with 0 RF (24 and 9 months vs. 47 months, respectively). Specifically, the favorable subgroup includes (a) PSA ≥ 20 ng/ml and clinical stage <cT2c and biopsy GS <8; (b) PSA <20 ng/ml and clinical stage =cT2c and biopsy GS <8; and (c) PSA ≥ 20 ng/ml and clinical stage =cT2c and biopsy GS <8. In other words, this patient group comprises the best candidates for RP while other groups are less likely to be treated with RP alone. Our preoperative risk stratification model of recurrence based on clinical T stage and biopsy Gleason score is simple to use in clinical practice. Given the increasing body of evidence about the efficacy of other treatment modalities such as EBRT + ADT, urologists and these patients should try to reach a shared clinically important decision making in terms of the optimal treatment option, taking into account patients' values and preferences, the complications profile, the financial costs, and the treatment modalities available on the urologist's armamentum. In contrast to advanced disease, preoperative inflammatory markers (NLR, dNLR, PLR, and LDH) are not of prognostic value in non-metastatic high-risk Pca patients in terms of BCR-free survival. Further and more powered studies are needed.

## Data Availability Statement

The raw data supporting the conclusions of this article will be made available by the authors, without undue reservation.

## Ethics Statement

The studies involving human participants were reviewed and approved by The Medical Ethics Committee of the Second Affiliated Hospital of Dalian Medical University. Written informed consent for participation was not required for this study in accordance with the national legislation and the institutional requirements.

## Author Contributions

GN performed the literature research, collected the data, and drafted the manuscript. GN and FT designed the research, performed data analysis, and interpretation. WS and SJ provided critical input in the research design, supervised the research, and the publication process. FT, WS, SJ, QW, and YW revised the manuscript. All authors have read and approved the final version of the manuscript.

## Conflict of Interest

The authors declare that the research was conducted in the absence of any commercial or financial relationships that could be construed as a potential conflict of interest.
